# The Treatment of Inoperable Cancer

**Published:** 1916-11

**Authors:** 


### The Treatment of Inoperable Cancer.

---

ABSTRACT OF REPORT OF TREATMENT BY DR. SILAS P. BEEBE, OF NEW YORK.

---

"The Treatment of Inoperable Cancer" is the title of a most interesting preliminary report devoted to a new treatment of malignant growths, by Silas P. Beebe, M. D., of 25 East Sixtieth Street, New York City, Professor of Experimental Therapeutics at the Cornell University Medical School and visiting physician to the General Memorial Hospital. Dr. Beebe estimates that 75,000 persons die annually in the United States from some form of cancer, and that at least 80 per cent of all persons suffering from malignant tumors, eventually reach the inoperable or incurable stage.

It is with some degree of trepidation that Dr. Beebe ventures to put before the medical profession a new method of treatment for inoperable cancer, and hopes his action will be judged solely on the data presented in his clinical report, as appeared in the *New York Medical Journal* of May 15, 1915.

Professor Beebe gives to Alexander Horovitz, Ph. D., an Austrian biologist and chemist, the credit of discovery; the technique and present development of this method having been evolved by Dr. S. P. Beebe and Dr. J. Wallace Beveridge.

Dr. Beebe in reporting his investigation of the patients treated at first, as examined by him before any were admitted to the General Memorial Hospital, stated that some of the patients had large, open, ulcerated tumors which previously had been the seat of active infective accompanied by the disagreeable odor associated with such a condition. Under this treatment, these infections were markedly influenced and the odor almost entirely disappeared, which impressed him sufficiently to warrant the method being given a thorough trial at the General Memorial Hospital.

The therapeutic agent employed in this treatment is a complex one, and it is believed that it has not been heretofore employed in the treatment of cancer. Dr. Beebe and Dr. J. Wallace Beveridge, working in conjunction with Dr. Horovitz, after many months of clinical experimentation, have been able to use the product as an extract administered subcutaneously. In referring to these clinical experiments, Dr. Beebe says: "At the point of injection in normal tissue, an active local reaction is produced; this reaction is evidenced by swelling, redness, heat, and tenderness. Then follows a general leucocytosis with a relatively high lymphocytosis, some rise in temperature, and, occasionally, a chill of varying intensity and duration. Now if the local area which receives the injection is examined microscopically, there are found all the characteristics of a moderately acute inflammatory reaction with a relatively large leucocytic infiltration. When such extracts were injected directly into a transplantable rat sarcoma, the characteristic reaction followed and was accompanied by a peculiar necrosis of the tumor cells with complete regression. When the skin over the tumor, prior to the injection, is ulcerated, the affected area rapidly degenerates and a mass of necrotic tissue is discharged, followed by healing, while if the skin is not broken or ulcerated, the reaction following the injection produced a marked infiltration of serum and leucocytes, particularly around the borders of the tumor, the tumor itself was gradually absorbed, and there was an apparent complete restoration of normal cellular conditions."

Subcutaneous injections of this extract have to a considerable extent displaced the direct tumor injection, having the obvious advantage of permitting a more certain dose, of bringing this therapeutic agent directly in contact with the growing border of the malignant cells, and producing in the depths of the tumor rather than on its surface an intense reaction, which appears to be unfavorable for the continued growth of the tumor. When these injections were first begun in human subjects, they were always confined to the growth itself. More recently they have been given subcutaneously in the arm, and it has been interesting to note that when so given there has been observed fairly definite reactive responses in the growth; these reactions in the growth are evidenced by swelling, temporary increase in pain, followed a few hours later by a considerable relief from pain, and in some forms of tumor by softening of the growth and a gradual diminution in its size.

Dr. Beebe's paper contains a report of two groups of cases, the first under his personal observation at the General Memorial Hospital, in which X-rays from a Coolidge tube formed a part of the treatment which was not so successful and satisfactory as in the second group of cases under the supervision of Dr. J. Wallace Beveridge at the Polyclinic Hospital. The latter group was treated entirely by hypodermic injection of the extract, no other therapeutic measures being used. The following two cases cited are taken from Dr. Beebe's preliminary report, viz.:

CASE XI.—Man, aged fifty years, had recurrent colloid carcinoma of the rectum. Kraske operation two years ago. In July recurrence became troublesome, and at the time of his admission to the hospital there was extensive involvement of the tissues, in and about the rectum and including the base of the bladder. Patient had severe pain, great difficulty in defecating. It was impossible at the time of his admission to pass a rectal tube, and the bladder irritation was so severe as to cause almost constant tenesmus. Injections at the hospital were made directly into the growth. During his stay in the hospital of six weeks, sixteen injections were made; following the earlier ones, there was marked reaction accompanied by rise of temperature and an occasional chill. X-ray examination revealed involvement of sacrum. Large broken down masses of tumor were discharged. Pain and irritation about the base of the bladder gradually diminished. The swelling and pain about the sacrum entirely disappeared, the tumor masses in the rectum were in part absorbed and in part broken down, and were discharged. At the end of six weeks the patient left the hospital, free from pain, with normal control of bladder. Patient was passing formed stools, the rectum admitted forefinger easily without pain, and tumor masses could not be felt. After leaving the hospital, that patient had a few injections in the arm, he continued to gain in weight and strength, and his general condition continued to improve.

CASE XV.—Woman, aged fifty-one years, had a recurrent inoperable carcinoma of the breast. Recurrence involved the fifth and sixth ribs, at the lower point of the old scar. Mass about three inches in diameter, very hard, the surface red. Some exudate appeared at the apex of the growth. Patient had intense pain along the left arm and shoulder, including the left side of the neck. Left arm markedly edematous and painful to touch. Edema extended to the region above the clavicle. Patient had had X-ray treatment for a short time prior to admission to hospital without effect. Treatment was entirely by injection into the tumor and into the arm on the right side. At the time of her admission and previously, patient had temperature of 100° to 101° F., which finally became normal. During the period of twenty-six days at the hospital, patient received eighteen injections, two being in the right arm. After her discharge, patient continued to receive the injections at weekly intervals into the right arm. When the patient left the hospital the induration had entirely disappeared, edema in the arm and above the clavicle had been absorbed, and she had no pain. The central portion of the tumor mass was marked by a scab, about a quarter of an inch in diameter, representing a point of an old sinus through which most of the tumor mass had been discharged. After she left the hospital, this area entirely healed, the patient returned to work and was subsequently entirely well.

Professor Beebe, in summing up his observation, states: "In spite of the somewhat complex and unusual character of the remedy employed, the evidence warrants further use of this method of treatment. In judging the merit of a treatment for inoperable cancer, it is probably wise to discount such matters as the relief from pain and the improvement of the general physical condition, because, while these matters are of much concern to the patient, they are to a considerable degree subjective in character and may to some extent be expected to follow the employment of any new method which stimulates the patient with faith and hope."

While Dr. Beebe has been interested in the treatment of cancer patients for a number of years, he has not heretofore seen such consistent improvement, of the character mentioned, follow in the type of patients cited by the use of other known remedies. If an actual diminution and regression in the bulk of malignant tissue is taken as a criterion by which to judge of the effect of this remedy, it is the writer's opinion that the evidence presented can lead to but one conclusion.

